# Optimization and economic evaluation of modified coagulation**–**flocculation process for enhanced treatment of ceramic-tile industry wastewater

**DOI:** 10.1186/s13568-018-0702-4

**Published:** 2018-10-17

**Authors:** Tahereh Zarei Mahmudabadi, Ali Asghar Ebrahimi, Hadi Eslami, Mehdi Mokhtari, Mohammad Hossein Salmani, Mohammad Taghi Ghaneian, Morteza Mohamadzadeh, Mohsen Pakdaman

**Affiliations:** 1 Environmental Science and Technology Research Center, Department of Environmental Health Engineering, School of Public Health, Shahid Sadoughi University of Medical Sciences, Pardis Campus, Gomnam Blv, Alem Squre, Yazd, 8915173160 Iran; 2 Department of Environmental Health Engineering, School of Health, Rafsanjan University of Medical Sceiences, Rafsanjan, Iran; 3 Environmental Science and Technology Research Center, Department of Statistics and Epidemiology, Shahid Sadoughi University of Medical Sciences, Yazd, Iran; 4 Environmental Science and Technology Research Center, Department of Management of Health Services, Shahid Sadoughi University of Medical Sciences, Yazd, Iran

**Keywords:** Coagulation, Flocculation, Poly**-**aluminum chloride, Polymer, Industrial wastewater

## Abstract

Enhanced treatment of ceramic-tile industry wastewater was investigated by modified coagulation–flocculation process using combination of poly**-**aluminum chloride (PAC) with anionic (A_300_), cationic polymer (C_270_) and nonionic polymers. The effects of pH, PAC coagulant dose alone and with polymers dose in various combinations was studied by jar tests. To compare the removal efficiencies of turbidity, total suspended solids (TSS), chemical oxygen demand (COD), and color at different levels, we run multivariate analysis of variance. Regarding the economic evaluation, we applied the incremental cost-effectiveness ratio. PAC had the best performance in pH 7 and in optimal dose of 400 mg/L; so that removal efficiency of wastewater turbidity, TSS, COD and color were 99.63%, 99.7%, 47.5% and 50.38%, respectively. The best removal efficiency for wastewater turbidity, TSS, COD and color were 99.87%, 99.89%, 87.5% and 93.02%, respectively which were obtained by combination of anionic polymer (1.5 mg/L) with PAC (300 mg/L). Furthermore, with combination of PAC + anionic + non-ionic polymers, the removal efficiency for wastewater turbidity, TSS, COD and color were 99.93%, 99.94%, 88% and 94.57%, respectively. The imposed cost for treating one cubic meter of ceramic-tile wastewater treatment by PAC + anionic and PAC + anionic and non-ionic polymers in comparison with PAC alone was reduced to 22.96% and therefore economically more affordable for the tile industry wastewater treatment.

## Introduction

Water is an important raw material in the ceramic-tile industries (Enrique et al. 2000; Gabaldón-Estevan et al. 2014). Water consumption varies in different parts and processes of manufacturing tiles (Huang et al. 2013). Generally, the consumed water per square meter of manufactured tile is about 20 L; 85 percent of this amount is consumed in the slurry process and the remaining 15 percent is used in the glazing section (Enrique et al. 2000). The wastewater of the ceramic-tile industries is produced through the following processes: slurring, spray drying, preparing glaze, coloring, engobing, as well as polishing and washing the floors of the production halls (Gabaldón-Estevan et al. 2014; Shu et al. 2010). The major part of the produced wastewater in these sectors is attributed to washing (Moliner-Salvador et al. 2012). Wastewaters of such industries contain clays, insoluble ferrites and silicates, electrolytes, anions such as sulfate (100–500 mg/L) and chloride (100–700 mg/L), as well as heavy metals such as lead, zinc, chemical oxygen demand (COD) (150–1000 mg/L), and BOD_5_ (50–400 mg/L). Organic materials in wastewater are mainly produced from the additives used in decorating tiles (Al-Asheh and Aidan 2017; Moliner-Salvador et al. 2012). In the ceramic-tile industries, a considerable amount of suspended solids and wastewater turbidity can be removed using a simple sedimentation process (Chong et al. 2009). The produced wastewater after this stage is only applicable in the slurry sector. This recycled water does not have the necessary quality to be used in other sectors, especially in glaze preparation or other coating processes. Therefore, it requires a more comprehensive treatment process (Chong et al. 2009; Martínez-García et al. 2012).

The wastewater of ceramic-tile industries can be treated using some techniques such as homogenization, aeration, sedimentation, filtration, adsorption by activated carbon, coagulation and flocculation, ion exchange and reverse osmosis (Al-Asheh and Aidan 2017; Radoiu et al. 2004). Although these are all effective methods, however, their major weakness is that they are expensive on a large scale (Eslami et al. 2018; Verma et al. 2010). Among these methods, coagulation and flocculation process is a suitable method for treatment of the municipal and industrial wastewater (Bakraouy et al. 2017; Simate et al. 2012; Teh et al. 2016).

Due to simple design and operation as well as low energy consumption, coagulation–flocculation process has been widely used for treatment of industrial wastewater (AlMubaddal et al. 2009). This method has been successfully applied in treatment of wastewater derived from industries such as tanneries (Haydar and Aziz 2009b), textile and dye (Sabur et al. 2012) paper and pulp mill (Kamali and Khodaparast 2015), petrochemical (Verma et al. 2010) and dairy industrials (Kushwaha et al. 2010). Coagulation–flocculation process initially involves addition of chemicals to destabilize the dissolved and suspended organic and inorganic pollutants. In the next stage, the destabilized pollutants are agglomerated to form folcs, which can be settled and removed from water and wastewater by sedimentation (Alexander et al. 2012; Teh et al. 2016). A large number of chemicals may be used as coagulants and flocculants in treating different types of wastewaters (Srivastava et al. 2005). These materials include inorganic and organic-based coagulants (Amuda and Amoo 2007, Lee et al. 2011). Poly aluminum chloride (PAC) is a mineral macro molecule in terms of composition and its monomers are dual-core complexes of aluminum (Wang et al. 2015). This compound forms multi-nuclear complexes in low concentrations and in aqueous media; this property has given PAC the unique ability to be applied in the process of coagulation and flocculation (Li et al. 2010). This coagulant has been extensively used in recent years and has become one of the most commonly applied coagulants in water and wastewater treatment in countries such as US, Canada, China, Italy, France, and UK (Ahmad et al. 2006; McCurdy et al. 2004). Operation in different pH ranges, low sensitivity to temperature, low residual in comparison to other metal coagulants, reduction of the sludge, and ease of sludge dewatering are among the benefits of PAC (Wang et al. 2015).

Polymers, conduct destabilizing activities through adsorption at the surface of colloidal particle and creating polymer-particle bridges (Hjorth and Jørgensen 2012; Lee et al. 2014). Polymers can be used as a coagulant aid to improve the performance of coagulants (Amuda and Amoo 2007; Lee et al. 2014). These coagulant aids build bridges between fine particles resulted from coagulants’ activities, create large and heavy clots, and accelerate the sedimentation process (Bolto and Gregory 2007). Polymers were studied in three anionic, cationic, and nonionic groups (Sahu and Chaudhari 2013) and one of their major benefits was reduction of the coagulant consumption (Radoiu et al. 2004). Some quantitative studies were conducted in the field of ceramic industries wastewater treatment by using coagulation and flocculation method. Chong et al. (2009) investigated the impact of adsorption—clotting mechanism on the removal of total suspended solids (TSS) and COD from ceramics’ industrial wastewater using palm oil mill boiler bottom ash as an absorbent and anionic polymer of 120c and cationic polymer of 1200B as flocculants. In another study, Hosseinzadeh (2011) which was investigated the impact of pH and concentration on the performance of cationic polymers and anionic acryl-amide in the treatment of ceramic industry wastewater.

However, the combined use of coagulants and polymers to increase the efficiency and reduce the costs is applied by researchers. Therefore, the aim of this study was to evaluate the PAC coagulant in combination with different types of polymers and also combination of polymers in removal of pollutants from wastewater, so that it can be used in production line of the ceramic-tile industry. Moreover, the economic evaluation of coagulation–flocculation process was investigated using incremental cost-effectiveness ratio (ICER).

## Materials and methods

### Sampling

Characteristics of the ceramic-tile industry raw wastewater are presented in Table 1. In this study, composite sampling was conducted from the production line wastewater considering the working shifts in the investigated ceramic-tile factory, Yazd, Iran. Parameters of pH, EC, and temperature were measured at the site. Principles of sampling, such as containers, sample size, storage, and retention time were obtained through the standard methods (APHA 2005). Specimens were stored at 4 °C after transferring to the laboratory.

**Table 1:** Characteristics of the raw wastewater tile industry

Parameter	Unit	Min	Max	Mean	S.D
PH	–	8.2	8.6	8.3	0.6
Temperature	(°C)	30	32	31 ± 1	1
Conductivity	(μs/cm)	2142	2700	2484	299.57
Turbidity	NTU	9500	13,300	11,100	1969.77
TDS	mg/L	1096	1266	1192	87.1
TSS	mg/L	14,300	34,414	21,638	11,105.05
COD	mg/L	151.2	490	361.3357	183.66
BOD_5_	mg/L	100.8	392.5	266.5167	149.58
Color	Pt-Co	219	300	217	41.5

*S.D* standard deviation

### Chemicals

Poly-aluminum chloride (Al_2_(OH)_n_Cl_6−n_. YH_2_O, Al_2_O_3_ = 30%wt, basicity = 65% and pH = 3) was used as a coagulant and anionic (A_300_), cationic (C_270_) and nonionic polymers, as the coagulant aids were provided from AquaTech Company, Switzerland.

### Preparation of solution

In order to prepare a 10 percent clear solution for coagulant, 10 g of PAC was dissolved to 100 mL of distilled water (EC = 0.1 µs/cm). In order to prepare a 0.1 percent solution as coagulant aid, 0.1 g of each polymer was provided separately. Considering the polymers, a 0.1 percent solution was prepared; 0.05 g of each polymer was separately dissolved into 100 ml of distilled water at temperature of 30–50 °C (Haydar and Aziz 2009a). Prepared polymer solutions were agitated at about 200–300 rpm by a shaker until the polymer particles were completely dissolved.

### Experimental procedure

**Fig. 1:**
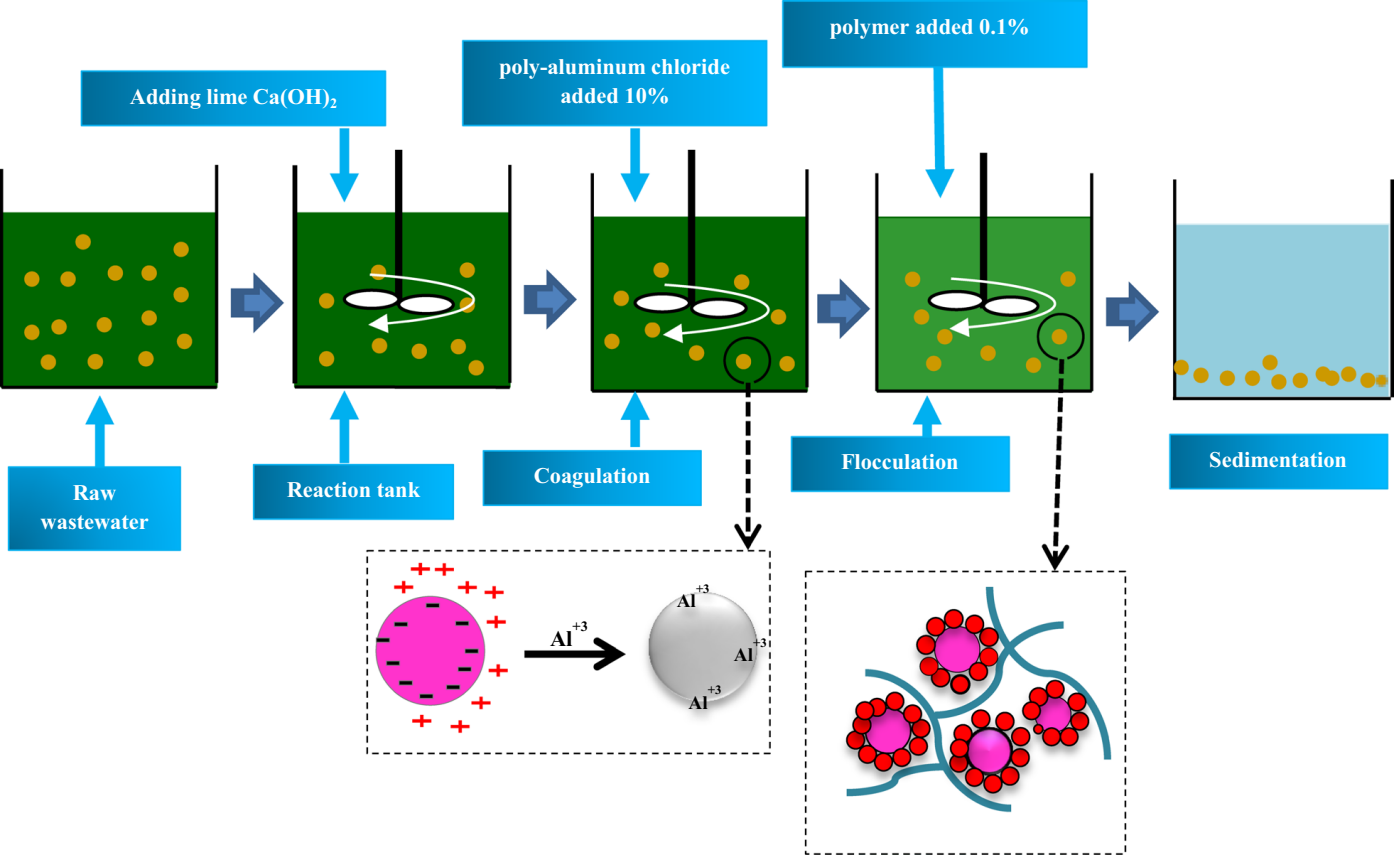
Schematic the coagulation and flocculation process

This study was conducted in the laboratory scale using a jar test manufactured by HACH Company of America (402-7790 model). All experiments were carried out at a temperature of 25 °C. In order to determine the best sedimentation time, 1 L of the wastewater samples was poured into an Erlenmeyer flask at sedimentation time of 10–120 min. Later, the wastewater turbidity and TSS removal were measured to determine the best time of sedimentation before coagulation and flocculation. In order to determine the optimal pH, we used the lime solution and normal hydrochloric acid and adjusted the pH in the range of 5–11. Then, the constant concentration of PAC (200 mg/L) was added to them using the jar test. The optimum pH was determined for each sample by measuring the removal efficiencies of turbidity, TSS, COD, and color (Ghafari et al. 2010). In the next step, different concentrations of PAC (150, 200, 250, 300, 350, 400 mg/L) were added to the samples to determine the optimum coagulant dose. Different polymer concentrations (0.5, 1, 1.5, 2, 2.5, 3 mg/L) were added to the samples separately and combinatorially and their optimal amounts were determined. All the experiments were performed on the same terms as the previous step and the optimum concentration was determined according to the studied parameters. Figure 1 illustrates the schematic process of coagulation and flocculation in this study. To increase the accuracy, all the experiments were repeated three times and the calculated mean values were reported as the final result.

### Physico-chemical analysis

Physicochemical parameters of the studied wastewater such as pH, EC, temperature, turbidity, total suspended solids (TSS), chemical oxygen demand (COD), biochemical oxygen demand (BOD_5_) and color were analyzed by using standard methods (APHA 2005). Parameters of PH and EC were measured by using multi-parameter (HACH-HQ40, US). Turbidity was measured by using turbidity meter (Eutech TB100) based on nephelometric turbidity unit (NTU). Methods of 5220-D and 5210-B were also applied to determine COD and BOD_5_. COD concentrations were measured by using potassium dichromate method. TSS and color were measured by spectrophotometry (DR 2000, HACH) (APHA 2005).

### Statistical and cost-effective analysis

Data normality was investigated by Kolmogorov–Smirnov and the homogeneity of data was determined using Levene test. To compare the turbidity, TSS, COD, and color of wastewater with the independent variables such as PH and different concentrations, we run the multivariate analysis (MANOVA) (P = 0.05). The Tukey test was also used to conduct multiple average comparisons between the groups.

In order to evaluate the economic efficiency, we used the ICER statistical formula (Eq. 1). In this formula, the cost differences between the two interventions are divided by the differences of their effects (Gafni and Birch 2006; Gaziano et al. 2006).

1 $${\text{ICER}} = \frac{{\left( {{\text{C}}_{1} - {\text{C}}_{0} } \right)}}{{\left( {{\text{E}}_{1} - {\text{E}}_{0} } \right)}}$$

where C_1_ and C_0_ are the cost and E_1_ and E_0_ are the effects in the intervention and control groups, respectively.

## Results

### Effect of primary sedimentation time

**Fig. 2:**
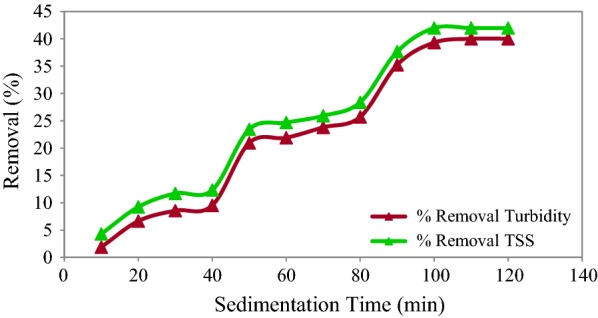
The effect of sedimentation time, before of coagulation process on removal turbidity and TSS

The effects of the primary sedimentation time on the TSS and turbidity removal efficiencies before adding a coagulant are represented in Fig. 2. As it shows, the removal efficiency remained fairly constant in the sedimentation time of 100 min. At this time, the turbidity reduced from 10,500 to 6310 NTU (39.9% removal efficiency) and TSS reduced from 15,750 to 9400 mg/L (41.97% removal efficiency).

### Effect of initial pH

**Fig. 3:**
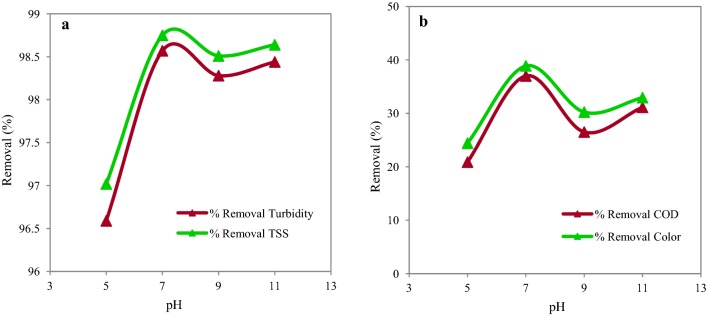
Removal efficiency of turbidity, TSS (**a**) and COD, color (**b**) in different pH (PAC = 200 mg/L)

The results of determining the optimal pH using a fixed dosage of PAC (200 mg/L) are represented in the Fig. 3. According to the results, the maximum removal efficiency of turbidity, TSS, COD and color were 98.57%, 98.75%, 37% and 38.37% respectively, which were observed at pH 7. The results of MANOVA and Wilks’ Lambda tests indicated that the pH level variable had a significant difference with at least one of the parameters of turbidity, TSS, COD, and color (P ≤ 0.001). Furthermore, the results revealed that the removal of turbidity, TSS, COD, and color in pH 7 had a significant difference with other pH levels.

### Effect of coagulant dosage without coagulant aid

**Fig. 4:**
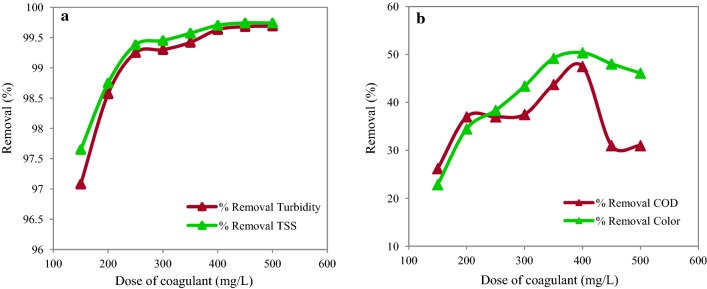
Removal efficiency of turbidity, TSS (**a**) and COD, color (**b**) for different doses of coagulant from wastewater (at optimum pH = 7)

The effects of PAC dosage on the removal efficiency of turbidity, TSS, COD and color is shown in the Fig. 4. According to the findings, the maximum removal efficiency was observed at the dose of 400 mg/L. In this PAC optimal dosage, the removal efficiencies of turbidity, TSS, COD, and color were 99.63%, 99.7%, 47.5% and 50.38%, respectively. As Fig. 4 illustrates, the turbidity, TSS, COD, and color removal efficiencies improved with the increase of the PAC dosage up to 400 mg/L. Regarding higher doses (> 400 mg/L), turbidity and TSS removal efficiencies were stable; whereas, the COD and color removal efficiencies decreased. The Wilks’ Lambda test showed that the doses of PAC were significantly different from at least one of the studied parameters (P ≤ 0.001). The results of the Tukey test indicated that different concentrations of PAC at the dose of 400 mg were significantly different with other doses regarding the removal of turbidity, TSS, COD, and color (P ≤ 0.001).

### Effect of coagulant dose with coagulant aid

In this stage of experiment, a constant dose of each polymer (0.5 mg/L) was added to different doses of PAC coagulants. The results are presented in Table 2. Based on these results, the highest removal efficiency in combinations of PAC (300 mg/L) with anionic polymer (A_300_) (0.5 mg/L) was observed 99.66% for turbidity, 99.72% for TSS, 85.12% for COD and 87.2% for colors. The best removal efficiency in combination of PAC (300 mg/L) + cationic polymer C_270_ was observed in 99.58% for turbidity, 99.64% for TSS, 52.5% for COD and 62.4% for color respectively. The highest removal efficiency in combination of 300 mg/L of PAC with non-ionic polymer were 99.41%, 99.48%, 76.25% and 55.03% for turbidity, TSS, COD and color, respectively. As a result, the applied polymers as coagulant aids decreased the efficient dose of PAC coagulants from 400 mg/L to 300 mg/L.

**Table 2:** The effects of anionic, cationic and nonionic polymer (0.5 mg/L) at different doses of PAC on the tile wastewater (at optimum pH = 7)

Polymers	Parameter	Dose of PAC (mg/L)					
		150	200	250	300	350	400
Anionic polymer	Turbidity (%)	99.11	99.31	99.54	99.66	99.66	99.65
	TSS (%)	99.3	99.44	99.61	99.72	99.72	99.72
	COD (%)	67.75	78.12	78.75	85.12	81.15	75.5
	Color (%)	55.42	67.05	79.45	87.2	87.2	87.2
Cationic polymer	Turbidity (%)	99.03	99.23	99.46	99.58	99.55	99.55
	TSS (%)	99.14	99.32	99.52	99.64	99.6	99.6
	COD (%)	37.5	41.25	47.47	52.5	51.5	49.9
	Color (%)	48.83	53.87	59.3	62.4	61.62	61.62
Nonionic polymer	Turbidity (%)	98.85	99.06	99.28	99.41	99.39	99.33
	TSS (%)	99	99.18	99.37	99.48	99.46	99.42
	COD (%)	59.7	64	65	76.25	75.5	65
	Color (%)	43.41	48.44	51.93	55.03	55.03	53.1

### Effects of polymers dose

**Fig. 5:**
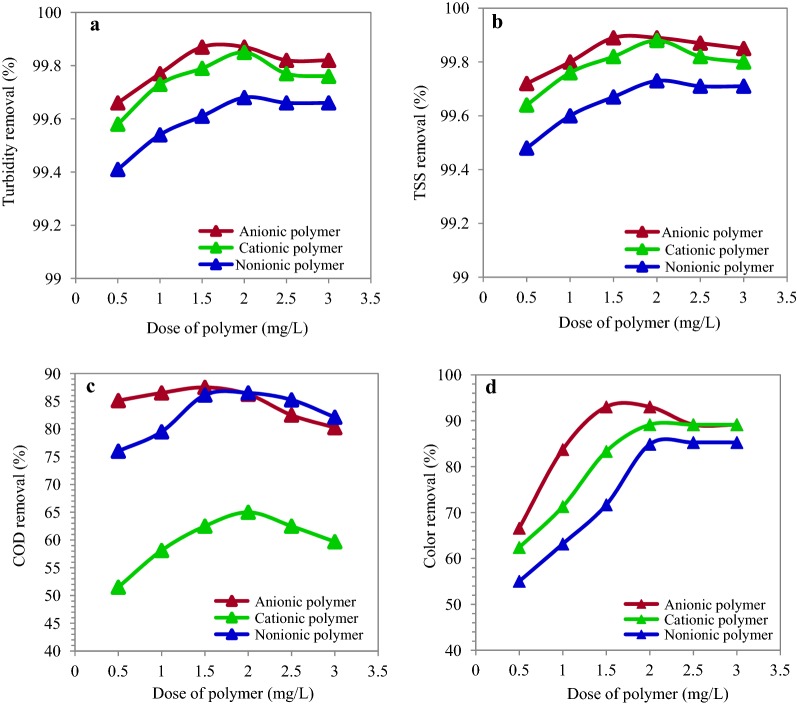
Turbidity (**a**), TSS (**b**), COD (**c**) and color (**d**) removal efficiency in different dose of anionic, cationic and nonionic polymers (At pH = 7 and PAC = 300 mg/L)

The purpose of this stage of jar-test experiment was to identify the suitable dose of polymers, which could be used in combination with optimal dose of PAC coagulant (300 mg/L) for treatment of the ceramic wastewater. The applied polymer dose varied from 0.5 to 3 mg/L. The effect of various doses of anionic A_300_, cationic C_270_, and non-ionic polymers on removal efficiency of turbidity, TSS, COD, and color are represented in Fig. 5. According to the results, the best removal efficiency for anionic A_300_ was 1.5 mg/L with removal efficiency of turbidity, TSS, COD and color 99.87%, 99.89%, 87.5% and 93.02%, respectively. The most removal efficiency was in 2 mg/L cationic polymer for turbidity (99.85%), TSS (99.88%), COD (65%) and color (89.14%). Furthermore, the most removal efficiency of non-ionic polymer for turbidity, TSS, COD and color were 99.68%, 99.73%, 86.5% COD and 84.88%, respectively, in concentration of 2 mg/L. As it is observed in Fig. 5, increase of the polymers’ concentration to an amount higher than the optimal dose decreased the removal efficiencies. The Wilks Lambda statistical test showed that the variable level of anionic, cationic, and non-ionic polymers had a significant difference with at least one of the wastewater parameters (P ≤ 0.001). The results of Tukey test indicated a significant difference among the various concentrations of anionic, cationic, and non-ionic polymers in removal of turbidity, TSS, COD, and color (P ≤ 0.001). This difference was higher for anionic polymer in the concentration of 1.5 mg and for cationic and non-ionic polymers in concentration of 2 mg/L.

### Effects of the polymers combination

**Fig. 6:**
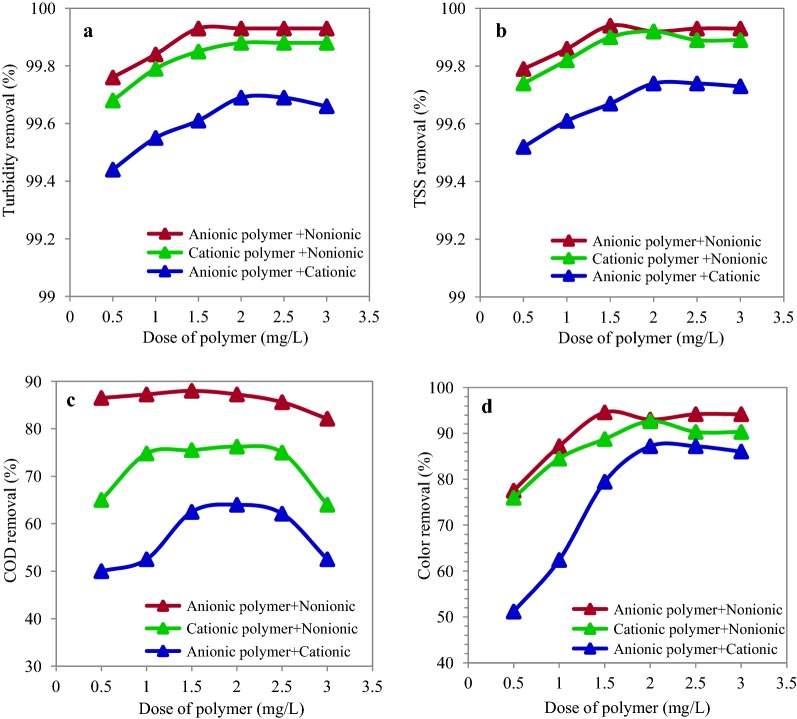
Turbidity (**a**), TSS (**b**), COD (**c**) and color (**d**) removal efficiency in different dose of combined polymers (at pH = 7 and PAC = 300 mg/L)

At this stage of the experiment, the anionic, cationic, and non-ionic polymers were combined in different doses of 0.5–3 mg/L. Later, we investigated the effect of this composition combined with the optimal dose of PAC. The effect of various doses of anionic-nonionic polymer combination on removal efficiencies of turbidity, TSS, COD, and color is shown in Fig. 6. According to the results, the highest removal efficiencies for turbidity, TSS, COD, and color in concentration of 1.5 mg/L of anionic-nonionic polymers’ combination were 99.93%, 99.94%, 88%, and 94.19%, respectively. In addition, regarding the cationic-nonionic polymer combination, the highest removal efficiencies observed in concentration of 2 mg/L were 99.88%, 99.92%, 76.25% and 92.63%, respectively. Moreover, the highest removal efficiencies for the cationic-anionic polymers combination observed in 2 mg/L concentration were 99.69%, 99.74%, 64% and 87.2%, respectively. The Wilks Lambda statistical test showed that the variable level of the polymers’ combination had a significant difference with at least one of the wastewater parameters including turbidity, TSS, COD, and color (P ≤ 0.001). The results of Tukey tests also showed a significant difference among various concentrations of the combined polymers in removal of turbidity, TSS, COD, and color (P ≤ 0.001). This difference was higher for anionic polymer + nonionic polymers in the concentration of 1.5 mg as well as for cationic + nonionic polymers and cationic + anionic polymers in the concentration of 2 mg.

### Economic evaluation

**Fig. 7:**
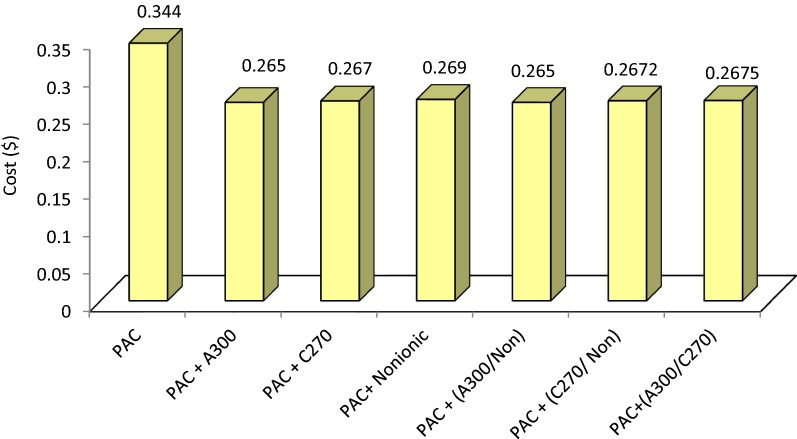
Cost of different options of coagulant—polymers for treatment of one cubic meter of ceramic tile wastewater

The comparisons of removal efficiencies attributed to different coagulant-polymers combinations are indicated in Table 3. The removal efficiencies of turbidity, TSS, COD, and color for PAC + (A_300_/nonionic) and PAC + A_300_ were higher than those of other options. The economic evaluation formula (ICER) was applied to compare the costs of different methods used for ceramic- tile wastewater treatment; the results of which are tabulated in Table 4. In addition, Fig. 7 illustrates the costs’ comparison of different methods used for ceramic-tile wastewater treatment.

**Table 3:** Comparison of Turbidity, TSS, COD and color removal efficiency for different combined coagulant-polymers options

Combined coagulant-polymers	Removal efficiency (%)			
	Turbidity	TSS	COD	Color
PAC + A_300_	99.87	99.89	87.5	93.02
PAC + C_270_	99.85	99.88	65	89.14
PAC + nonionic	99.68	99.73	86.5	84.88
PAC + (A_300_/non)	99.93	99.94	88	94.19
PAC + (C_270_/non)	99.88	99.92	76	76
PAC + (A_300_/C_270_)	99.69	99.74	64	87.2

**Table 4:** ICER estimated for compounds used for ceramic-tile wastewater treatment

Combined coagulant-polymers	Removal efficiency (%)	Cost ($)	ICER
PAC + A300	83.07	− 0.0787	− 0.0009474
PAC + C_270_	56.66	− 0.0768	− 0.0013555
PAC + nonionic	73.35	− 0.075	− 0.0010193
PAC + (A_300_/non)	85.23	− 0.0789	− 0.0009257
PAC + (C_270_/non)	71.47	− 0.0768	− 0.0010746
PAC + (A_300_/C_270_)	13.355	− 0.0765	− 0.001432

### Heavy metal and boron removal efficiency

**Fig. 8:**
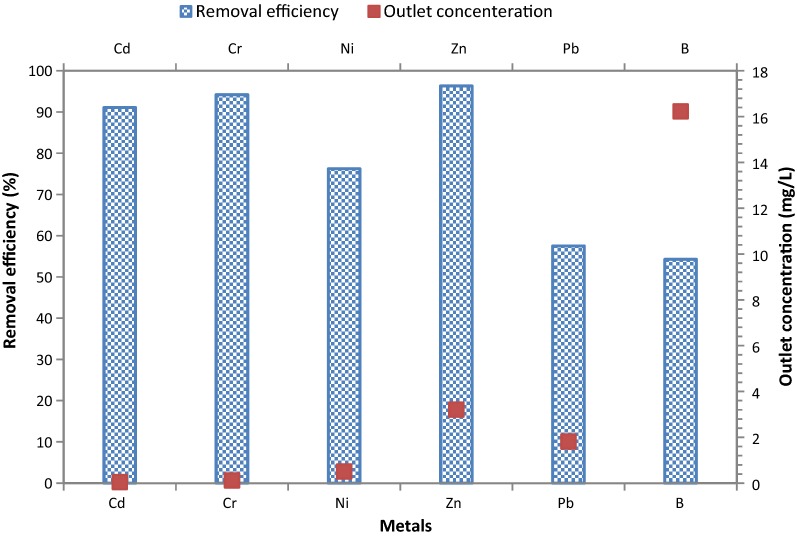
Removal efficiency and outlet concentration of heavy metal and born in the coagulation–flocculation process (at pH = 7 and PAC = 300 mg/L, anionic + nonionic polymers = 1.5 mg/L)

Removal efficiency of heavy metals including cadmium (Cd), chromium (Cr), nickel (Ni), zinc (Zn), lead (Pb), and boron (B) in the coagulation–flocculation process under the optimum condition are represented in Fig. 8. The mean removal efficiencies for Cd, Cr, Ni, Zn, Pb, and B were 91.11%, 94.23, 76.25%, 96.32%, 75.53% and 54.28%, respectively.

## Discussion

In this study, the TSS and turbidity removal efficiencies at the primary sedimentation time of 100 min were 39.9% and 41.97%, respectively. Fahimnia et al. (2013) studied the stone cutting industry wastewater treatment by coagulation and similarly found that the optimum primary sedimentation time of wastewater was 100 min before the coagulation process. Turbidity and TSS removal efficiencies in the primary sedimentation can be due to high concentration of suspended solids in the raw wastewater (Chong et al. 2009).

The maximum removal efficiency of turbidity, TSS, COD, and color was observed at pH 7. In the process of coagulation and flocculation, pH is a very important factor that effects the hydrolysis balance (Verma et al. 2012). The main reason for better performance of PAC at pH 7 is that the Al hydroxide flocs species are charged positively in pH rates of 5–7 (Al(OH)_2_^+^ and Al(OH)^2+^). So, they neutralize the charges, adsorb the organic pollutants and solids, and consequently increase the removal efficiency (Yang et al. 2010). At low pH, concentration of the dissolved aluminum decreases by reduction of the Al (OH)_4_^−^ ratio. Reduction of this ratio improves the sedimentation process and the anionic aluminum hydroxide reduces the clotting effects (Wang et al. 2015). Moreover, we found that the rate of COD removal reduced in alkaline pH. This can be due to the formation of neutralized species of Al hydroxide flocs (Al(OH)_3_), in which charge neutralization and adsorption mechanisms did not happen for the pollutant removal (Yang et al. 2010). In general, the pH solution affects the production capacity of hydroxyl flocs. Ghafari et al. (2010) investigated the effect of PAC on the emulsion treatment. Their results showed that the coagulant at pH of 5–7 had the best performance for turbidity, TSS, COD, and color that was close to the optimum pH calculated in our study.

In the present study, the optimal PAC dose was 400 mg/L. These findings indicate that high doses of PAC are needed for treatment of the ceramic industrial wastewater. This may be due to the presence of large amounts of organic matters in wastewater and their reaction to coagulants, which decreased the removal efficiency (Matilainen et al. 2010). Furthermore, the highest removal efficiency was observed in combinations of PAC with anionic polymer (A_300_). As a result, application of polymers as coagulant aids decreased the efficient dose of PAC coagulant. Polymers act as aids in cleansing the water and wastewater. They even can be used as primary coagulants for some purposes (Zhao et al. 2012). According to the results, the best removal efficiency for turbidity, TSS, COD and color was observed for anionic A_300_ polymer in concentration of 1.5 mg/L and for cationic and non-ionic polymers in concentration of 2 mg/L. In addition, increase of the polymers’ concentration to a rate higher than the optimal dose decreased the removal efficiency. In the same regard, Chong et al. showed that increase of the anionic C120 and cationic B120 polymers decreased the removal efficiency of TSS (Chong et al. 2009). This can be justified by the fact that increase of the polymer dose results in resuspension of flocs and this in turn decreases the removal efficiency (Saritha et al. 2017).

The effects of anionic, cationic, and non-ionic polymers’ combination and their application along with the PAC showed that the best removal efficiency was observed in PAC + A_300_ and PAC + A_300_/nonionic combination. A review on the available resources indicated that little information exists about toxicity of polymers (Ji et al. 2017). Generally, the cationic polymers seem to be more poisonous than other polymers. Therefore, the sludge containing the poisons in the form of aluminum and cationic polymers should be treated with more caution (Liber et al. 2005).

Results of the economic evaluation achieved by ICER showed that the combination of PAC + A_300_ and PAC + A_300_/nonionic was more economical for treating one cubic meter of ceramic-tile wastewater in comparison with other choices and reduced the cost up to 22.96 percent. A study on the tannery wastewater treatment using the coagulation–flocculation method showed that the combination of alum with cationic polymers was more efficient and economical than the combination of alum with anionic polymers. It was also reported in this research that by application of the alum-cationic polymer combination, the cost for treatment of one cubic meter of wastewater reduced 50 percent compared with application of alum–alone method (Haydar and Aziz 2009b). However, in the current study, we found that the combination of PAC with aniconic and nonionic polymers was more efficient. Al(OH)_3_ hydroxide flocs with positive charge can adsorb anionic polymers, form larger flocs, bridge between the hydroxide flocs, and adsorb organic and colloidal materials in sweep flocculation process. Therefore, the removal efficiency for turbidity, TSS, COD, and color increased; whereas, the cost of treatment reduced (Haydar and Aziz 2009b; Yang et al. 2010).

The highest removal efficiencies of heavy metals and B from tile industry wastewater were respectively attributed to Zn, Cr, Cd, Ni, Pb, and B using the coagulation–flocculation process by PAC + anionic and non-ionic polymers. The main mechanism for removal of heavy metals and B included surface adsorption of heavy metals on the Al hydroxide clusters along with polymers and their simultaneous sedimentation (Fu and Wang 2011; Hargreaves et al. 2018). The coagulants are effective receptors of heavy metals because they form a series of water-soluble multi-capacity metal ions, which absorb and then remove the heavy metals (Fu and Wang 2011). The high removal efficiencies of Zn, Cr, and Cd can be due to the colloidal size fraction of Zn and the particulate fraction of the Cr and Cd distribution in the wastewater. As a result, the risk of collision with the clots formed in the coagulation–flocculation process increases and their removal efficiencies improve (Hargreaves et al. 2018).

Finally, the results of this study show that the coagulation–flocculation process as well as the PAC combination with anionic, cationic, and nonionic polymers can be used as an effective method for treatment of ceramic-tile wastewater. The PAC, as the coagulator with the optimal dose of 300 mg/L and the anionic polymer A_300_, as the coagulant aid with the optimal dose of 1.5 mg/L in the normal pH of wastewater (pH = 7) showed the highest removal efficiency for turbidity, TSS, COD, and color. Considering the polymers’ combinations, the combination of anionic A300 and non-ionic polymers in the optimal dose had the highest removal efficiency. The treatment cost for one cubic meter of ceramic-tile wastewater using the PAC + anionic as well as PAC + anionic and non-ionic polymers was 22.96 percent less than the PAC-alone method.

## Abbreviations

TSS: total suspended solids
COD: chemical oxygen demand
BOD_5_: biochemical oxygen demand
EC: electrical conductivity
ICER: incremental cost-effectiveness ratio
MANOVA: multivariate analysis of variance
PAC: poly aluminum chloride
